# Electrophysiology Team–Administered Conscious Sedation for Pulsed‐Field Versus Radiofrequency Ablation in Atrial Fibrillation

**DOI:** 10.1155/anrp/4197770

**Published:** 2026-10-29

**Authors:** Li Cheng, Yu Xia, Zhicheng Hu, Tao Zhang, Le Li, Xu Meng, Yulong Xiong, Qin Xu, Zhenhao Zhang, Guanzhi Chen, Ligang Ding, Yan Yao

**Affiliations:** ^1^ Department of Cardiology Beijing Tiantan Hospital Capital Medical University Beijing China ccmu.edu.cn; ^2^ Cardiac Arrhythmia Center Fuwai Hospital National Key Laboratory National Center for Cardiovascular Diseases Chinese Academy of Medical Sciences and Peking Union Medical College Beijing China cacms.ac.cn

**Keywords:** atrial fibrillation, conscious sedation, pulsed-field ablation

## Abstract

**Background:**

Pulsed‐field ablation (PFA) is emerging as a novel nonthermal alternative. However, PFA is commonly performed under general anesthesia or deep sedation requiring anesthesiologists, which increases resource utilization and limits its widespread application. This study aimed to evaluate the feasibility of conscious sedation for PFA administered by the electrophysiology (EP) team, using radiofrequency ablation (RFA) as a comparator.

**Methods:**

A total of 120 patients with symptomatic, drug‐refractory atrial fibrillation were randomized in a 1:1 ratio to the PFA group or the RFA group. We evaluated three key outcomes: (1) intraoperative patient tolerance, assessed using validated scales (Faces Pain Scale‐Revised [FPS‐R], Likert Scale Questionnaire [LSQ]) combined with intraoperative body movement and cough reactions; (2) procedural efficiency; and (3) procedural safety.

**Results:**

The incidence of pain during pulmonary vein isolation was comparable between the two groups (all *p* > 0.05), whereas FPS‐R scores were significantly lower in the PFA group (*p* < 0.05). No significant difference was observed in movement scores between the two groups (*p* = 0.452). According to the LSQ analysis, only 1 patient (1.7%) in the RFA group reported dissatisfaction with intraoperative pain management. Cough incidence during PVI was higher with PFA (21.7% [13/60] vs 6.7% [4/60]; *p* = 0.018). Both total procedure time and left atrial dwell time were shorter in the PFA group. Furthermore, no serious complications were directly related to conscious sedation.

**Conclusion:**

In selected low‐risk patients, EP team–administered conscious sedation is feasible for PFA, with acceptable intraoperative tolerance and safety while preserving procedural efficiency.

## 1. Introduction

Atrial fibrillation (AF) is one of the most common clinical arrhythmias, and catheter ablation has become an important treatment strategy for symptomatic AF [1, 2]. Traditionally, radiofrequency ablation (RFA) has been the mainstream thermal ablation technique. However, it causes tissue damage through thermal effects, which carries risks such as injury to adjacent structures and intraoperative pain for patients [3–5].

Pulsed‐field ablation (PFA) is an emerging nonthermal ablation technology. It selectively targets cardiomyocytes through the principle of irreversible electroporation, offering significant theoretical advantages including high tissue specificity, shorter procedure times, and avoidance of thermal injury–related complications [6, 7]. PFA is rapidly becoming a new mainstream approach for AF ablation. Nevertheless, PFA delivery can potentially trigger severe chest pain, coughing, and muscle contractions, leading most centers currently to adopt general anesthesia (GA) to ensure procedural stability [8–10]. While GA provides ideal intraoperative conditions, its reliance on anesthesiologist resources, longer preprocedural preparation and postprocedural recovery times, and higher overall costs limit its widespread application in regions with relatively constrained healthcare resources [11, 12]. Some investigators have attempted to use deep sedation administered by anesthesiologists as a compromise, yet this still does not fundamentally resolve the dependency on specialized anesthesia personnel [13, 14].

Against this backdrop, exploring a more streamlined conscious sedation protocol holds clinical and practical importance. This study compared intraoperative tolerance, procedural efficiency, and safety between PFA and RFA under a standardized conscious sedation protocol, aiming to evaluate the feasibility of EP team–administered conscious sedation for PFA in selected low‐risk patients.

## 2. Methods

### 2.1. Study Population and Grouping

This study prospectively enrolled patients with symptomatic, drug‐refractory AF scheduled for catheter ablation between May 2025 and September 2025. Patients were randomized 1:1 to PFA or RFA using block randomization, with the random sequence generated by an independent statistician and allocation concealed via sequentially numbered opaque sealed envelopes.

Exclusion criteria were as follows: (1) obstructive sleep apnea and difficult airway (Mallampati Grade III/IV); (2) New York Heart Association Class III–IV heart failure; (3) structural heart disease or severe valvular disease; (4) acute myocardial infarction, stroke, or major bleeding within 3 months; (5) morbid obesity with BMI ≥ 35 kg/m^2^; (6) long‐term psychiatric disorder, history of substance abuse, or narcotic drug misuse; and (7) glaucoma or contraindications to the use of related anesthetic medications. All study participants provided informed consent. The study was approved by the Ethics Committee of Fuwai Hospital, Chinese Academy of Medical Sciences.

### 2.2. Conscious Sedation Protocol and Procedure

Conscious sedation was administered by the EP team, with nurses specially trained in clinical anesthesia. The primary operator and first assistant maintained continuous communication with the patient to evaluate sedation depth. An anesthesiologist was available on call. The protocol is schematically illustrated in (Figure 1).

**FIGURE 1:**
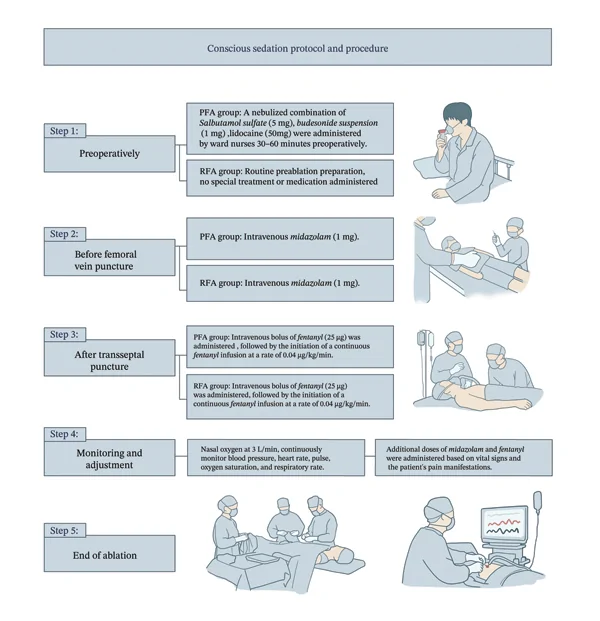
Conscious sedation protocol for pulsed‐field ablation and radiofrequency ablation. The protocol outlines the step‐by‐step sedation regimen, preoperative preparation, intraoperative monitoring, and adjustment strategies for both PFA and RFA procedures, including medication administration timing, dosage, and monitoring parameters. Abbreviations: PFA, pulsed‐field ablation; RFA, radiofrequency ablation.

PFA Group Sedation Protocol:

1. Preoperatively: A nebulized combination of salbutamol sulfate (5 mg), budesonide suspension (1 mg), and lidocaine (50 mg) was administered by ward nurses 30 min preoperatively.
2. Before femoral vein puncture: intravenous midazolam (1 mg)
3. After transseptal puncture: intravenous bolus of fentanyl (25 μg) was administered, followed by the initiation of a continuous fentanyl infusion at a rate of 0.04 μg/kg/min.
4. Continuous monitoring and titration of midazolam/fentanyl as needed.

RFA Group Sedation Protocol:

1. Before femoral vein puncture: intravenous midazolam (1 mg)
2. After transseptal puncture: intravenous bolus of fentanyl (25 μg) was administered, followed by the initiation of a continuous fentanyl infusion at a rate of 0.04 μg/kg/min.
3. Continuous monitoring and titration of midazolam/fentanyl as needed.

Both groups received nasal oxygen at 3 L/min. Blood pressure, heart rate, oxygen saturation, and respiratory rate were continuously monitored.

### 2.3. Ablation Procedure

All procedures were performed by highly experienced electrophysiologists. The PFA group used the VARIPULSE catheter (Biosense Webster) with the CARTO3 system, while the RFA group used the ST‐SF RFA catheter (Biosense Webster) with CARTO3. Transseptal puncture was performed under fluoroscopic or intracardiac echocardiography guidance. Standard electroanatomical mapping was performed in all patients before and after ablation. All patients underwent pulmonary vein isolation (PVI); supplemental ablation was performed at the operator’s discretion based on individual substrate assessment.

### 2.4. Outcome Assessments

The primary endpoint was intraprocedural pain during PVI, assessed by Faces Pain Scale‐Revised (FPS‐R) scores. Secondary endpoints included (1) other tolerance outcomes, (2) procedural efficiency, and (3) periprocedural safety. The definitions and criteria are detailed in Supplement 1.

#### 2.4.1. Intraoperative Patient Tolerance Assessment

Patient intraoperative tolerance was assessed using validated scales, intraoperative body movement grading, and cough reactions. Richmond Agitation‐Sedation Scale (RASS) scores, body movement grade, and cough reactions were jointly evaluated by the primary operator and first assistant. The Likert Scale Questionnaire (LSQ) was completed by patients after the procedure, while FPS‐R scores were obtained via patient self‐report after each PVI.

• RASS: Used to assess the level of patient agitation and sedation, comprising 10 levels (−5 to + 4). A score of −5 indicates no response to any stimulus, while + 4 indicates combative behavior [15].
• FPS‐R: Rapid assessment of pain intensity; it utilizes 6 different facial expressions ranging from “No Pain” (0 points) to “Most Severe Pain” (10 points) [16].
• LSQ: Consists of several declarative items that express a viewpoint on a topic. Respondents can indicate the degree to which they agree or disagree with the opinion expressed by the statement [17].
• Intraoperative body movement assessment: Grade 0 (no movement), Grade 1 (mild movement: slight tremor of fingers, toes, or head, no impact on catheter stability), and Grade 2 (significant movement: trunk, upper/lower limb movement, requiring procedure suspension).

#### 2.4.2. Procedural Efficiency Assessment

To assess the procedural efficiency of AF catheter ablation under conscious sedation, the following intraoperative data were recorded: total procedure time (skin‐to‐skin time), left atrial (LA) catheter dwell time, first‐pass isolation (FPI) rate and isolation time of the pulmonary veins, and supporting ablation locations.

#### 2.4.3. Safety

To evaluate the safety of AF catheter ablation performed under conscious sedation, intraoperative transient adverse events, procedural complications, and sedation‐related interventions were documented. Transient adverse events included transient hypotension, hypoxemia, bradycardia, tachypnea, and phrenic nerve stimulation. Procedural complications comprised pseudoaneurysm, cardiac tamponade, phrenic nerve palsy, myocardial perforation, and esophageal or vagus nerve injury. Sedation‐related interventions included jaw thrust, assisted ventilation, administration of reversal agents, and conversion to GA.

### 2.5. Statistical Analysis

Sample size calculation for the between‐group comparison of means was performed with the FPS‐R score. Based on our pilot study data (PFA: 2.5 ± 1.2; RFA: 4.0 ± 1.5), a clinically relevant mean difference of 1.5 in FPS‐R scores between groups was used. With a two‐sided *α* of 0.05 and test power (1‐*β*) of 90%, the minimum required sample size was 51 patients per group. Accounting for an anticipated 10% data loss rate, the minimum final sample size was set at 56 patients per group, using PASS 21 software (NCSS, LLC, Kaysville, Utah, USA).

Continuous variables were expressed as mean ± standard deviation (SD) or median (interquartile range [IQR]). Categorical variables were presented as counts and percentages (%). Differences between continuous variables were analyzed using the independent samples *t*‐test (for normally distributed data) or the Mann–Whitney *U* test (for non‐normally distributed data). For categorical data, comparisons were performed using Pearson’s chi‐square (*X* ^2^) test or Fisher’s exact test, as appropriate.

A two‐tailed *p* < 0.05 was considered statistically significant. All analyses were conducted with IBM SPSS Statistics, Version 26.0 (IBM Corp., Armonk, NY, USA).

## 3. Results

### 3.1. Patient Baseline Characteristics

A total of 120 patients were enrolled. The mean (SD) age was 63.7 (10.6) years in the PFA group (29 male [48.3%]) and 62.2 (9.1) years in the RFA group (38 male [63.3%]). Baseline demographic and clinical characteristics were not significantly different between groups (Table 1).

**TABLE 1:** Baseline characteristics.

	PFA group	RFA group	*p* value
	*n* = 60	*n* = 60	
Age, mean (SD), years	63.7 (10.6)	62.2 (9.1)	0.420
Male, *n* (%)	29 (48.3%)	38 (63.3%)	0.098
BMI, mean (SD), kg/m2	25.2 (2.5)	25.3 (2.3)	0.856
Neck circumference, mean (SD), cm	49.1 (7.0)	48.2 (5.9)	0.465
Waist circumference, mean (SD), cm	96.1 (9.8)	95.5 (8.5)	0.737
Degree of obesity			0.437
Normal weight, *n* (%)	26 (43.3)	32 (53.3)	
Overweight, *n* (%)	30 (50.0)	23 (38.3)	
Obesity *n* (%)	4 (6.7)	5 (8.3)	
Initial ablation *n* (%)	52 (86.7)	47 (78.3)	0.230
AF duration, median (IQR), years	1.3 (0.6–3.7)	2.0 (0.9–4.0)	0.492
Type of AF			0.326
Paroxysmal AF, *n* (%)	38 (63.3)	36 (60.0)	
Persistent AF, *n* (%)	12 (20.0)	8 (13.3)	
Long‐standing persistent AF (%)	10 (16.7)	16 (26.7)	
CAD, *n* (%)	8 (13.3)	12 (20.0)	0.327
COPD, *n* (%)	2 (3.3)	0 (0.0)	
Mallampati grade I/II, *n* (%)	60 (100)	60 (100)	1.000
Diabetes mellitus, *n* (%)	18 (30)	10 (16.7)	0.084
Hypertension, *n* (%)	28 (46.7)	38 (63.3)	0.067
Heart failure, *n* (%)	2 (3.3)	4 (6.7)	0.402
Renal failure, *n* (%)	0 (0.0)	0 (0.0)	
LA diameter, mean (SD), mm	41.2 (3.5)	40.1 (3.4)	0.105
LA volume, median (IQR), mL	65.5 (59.0–83.1)	65.0 (55.0–75.0)	0.165
LVEF, mean (SD), %	65.4 (5.1)	63.6 (6.8)	0.096
CHA_2_DS_2_‐VASc score	2.0 (1.0–2.0)	2.0 (1.0–2.0)	0.786

*Note:* BMI, body mass index (calculated as weight in kilograms divided by height in meters squared); RFA, radiofrequency ablation.

Abbreviations: AF, atrial fibrillation; CAD, coronary artery disease; COPD, chronic obstructive pulmonary disease; LA, left atrial; PFA, pulsed‐field ablation.

### 3.2. Analysis of Intraoperative Tolerance

The overall median (IQR) RASS score was 0 (−1, 0) across both groups. 92% (110/120) of patients maintained between −1 and 0, while 8% (10/120) had a score of 1. Total doses of sedative agents were comparable between groups (midazolam: 2.8 [2.0–3.5] vs 2.5 [2.0–3.0] mg, *p* = 0.352; fentanyl: 167 (150–181) vs 173 (162–186) *μg*, *p* = 0.304) (Table 2).

**TABLE 2:** Procedural parameters and sedative doses.

	RFA group	RFA group	*p* value
	*n* = 60	*n* = 60	
Procedural duration, mean (SD), min	50.3 (10.3)	62.4 (11.1)	< 0.001
Catheter LA dwell time, mean (SD) min	36.9 (8.6)	52.4 (9.7)	< 0.001
LSPV first‐pass isolation rate, *n* (%)	57 (95.0)	47 (78.3)	0.016
Time to achieve PVI, median (IQR), min	5.0 (4.0–7.0)	10.0 (10.0–12.0)	< 0.001
LIPV first‐pass isolation rate, *n* (%)	60 (100)	50 (83.3)	< 0.001
Time to achieve PVI, median (IQR), min	5.0 (3.0–6.2)	10.0 (7.0–11.0)	< 0.001
RSPV first‐pass isolation rate, *n* (%)	56 (93.3)	43 (71.7)	0.002
Time to achieve PVI, median (IQR), min	5.0 (3.7–7.0)	10.0 (9.0–13.0)	< 0.001
RIPV first‐pass isolation rate, *n* (%)	59 (98.3)	49 (81.7)	0.002
Time to achieve PVI, median (IQR), min	4.5 (3.0–6.2)	8.0 (7.0–10.2)	< 0.001
Supporting ablation			
SVC, *n* (%)	44 (73.3)	18 (30.0)	< 0.001
BOX, *n* (%)	38 (63.3)	12 (20.0)	< 0.001
CTI, *n* (%)	9 (15.0)	6 (10.0)	0.792
MI, *n* (%)	24 (40.0)	8 (13.3)	0.011
AW, *n* (%)	0 (0.0)	2 (3.3)	0.862
SI, *n* (%)	6 (10.0)	0.0 (0.0)	0.201
Sedative agents			
Midazolam, median (IQR), mg	2.8 (2.0–3.5)	2.5 (2.0–3.0)	0.352
Fentanyl, median (IQR), μg	167 (150–181)	173 (162–186)	0.304

*Note:* BOX, posterior wall BOX ablation; CTI, cavotricuspid isthmus; RFA, radiofrequency ablation.

Abbreviations: AW, anterior wall; LIPV, left inferior pulmonary vein; LSPV, left superior pulmonary vein; MI, mitral isthmus; PFA, pulsed‐field ablation; RIPV, right inferior pulmonary vein; RSPV, right superior pulmonary vein; SI, septal isthmus; SVC, superior vena cava.

Pain incidence during PVI was comparable between groups (*p* > 0.05) (Figure 2A). However, the median [IQR] FPS‐R were significantly lower in the PFA group (LSPV: 2.0 [2.0–3.0] vs 4.0 [2.5–4.0], *p* = 0.030; LIPV: 2.0 [1.7–3.0] vs 3.5 [2.0–4.5], *p* = 0.039; RSPV: 2.0 [1.5–2.7] vs 4.0 [3.5–4.0], *p* = 0.010; RIPV: 2.0 [2.0–2.5] vs 4.0 [3.0–4.7], *p* = 0.006) (Figure 2B). For intraoperative body movement, the majority of patients were Grade 0 or Grade 1; only 6.7% of PFA patients and 3.3% of RFA patients showed Grade 2 movement, with no significant difference between groups (*p* = 0.452) (Figure 2C). Based on the analysis of the LSQ, only 1 patient (1.7%) in the RFA group reported dissatisfaction with intraoperative pain management (Figure 2D). Cough incidence during PVI was higher in the PFA group (21.7% [13/60] vs 6.7% [4/60]; *p* = 0.018) (Figure 3A), occurring most often during LSPV ablation (20.0%, [12/60]). In the exploratory analysis of supplemental sites, cough occurred during 14 of 48 posterior wall BOX applications (25.0%) (Figure 3B).

**FIGURE 2:**
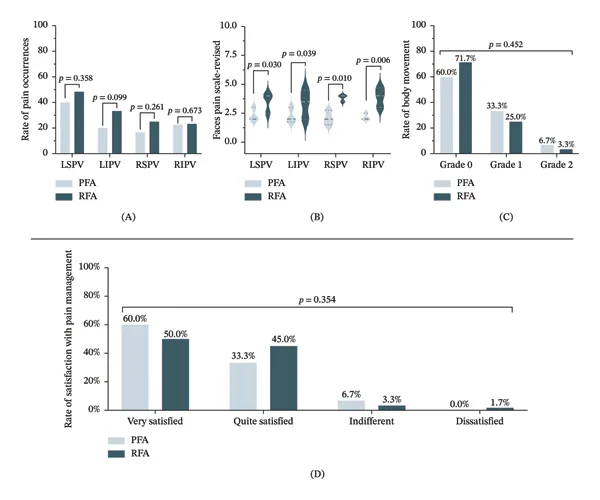
Comparison of intraoperative tolerance outcomes. (A), rate of pain occurrences during pulmonary vein isolation in PFA and RFA groups. (B), faces pain scale‐revised (FPS‐R) scores across pulmonary veins in PFA and RFA groups. (C), the intraoperative body movement rate by grade in PFA and RFA groups. (D), the Likert Scale Questionnaire assesses patients’ satisfaction with intraoperative pain management. *p* values denote the statistical significance between groups. LSPV, left superior pulmonary vein; LIPV, left inferior pulmonary vein; RSPV, right superior pulmonary vein; RIPV, right inferior pulmonary vein.

**FIGURE 3:**
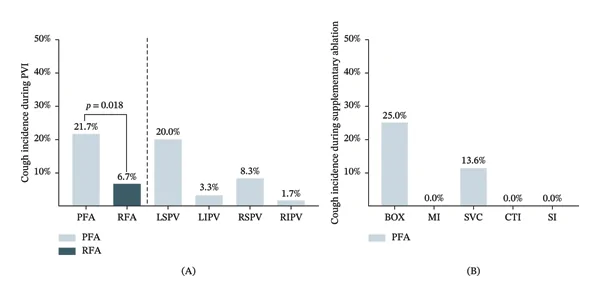
Cough incidence during PVI and supporting ablation. (A) Patient‐level cough incidence between groups and site‐specific distribution in the PFA group during PVI. (B) Cough incidence at supporting ablation sites in the PFA group. *p* value denotes the statistical significance between groups. BOX, posterior wall BOX ablation; CTI, cavotricuspid isthmus; LSPV, left superior pulmonary vein; LIPV, left inferior pulmonary vein; MI, mitral isthmus; PFA, pulsed‐field ablation; PVI, pulmonary vein isolation; RFA, radiofrequency ablation; RSPV, right superior pulmonary vein; RIPV, right inferior pulmonary vein; SI, septal isthmus; SVC, superior vena cava.

### 3.3. Analysis of Procedural Efficiency

The PFA group had significantly shorter mean (SD) total procedure time (50.3 [10.3] min vs 62.4 [11.1] min, *p* < 0.001) and catheter LA dwell time (36.9 [8.6] min vs 52.4 [9.7] min, *p* < 0.001) (Table 2). FPI of pulmonary veins in the PFA group was significantly higher than in the RFA group (LSPV: 95.0% [57/60] vs 78.3% [47/60], LIPV: 100% [60/60] vs 83.3% [50/60], RSPV: 93.3% [56/60] vs 71.7% [43/60], RIPV: 98.3% [59/60] vs 81.7% [49/60]; all *p* < 0.05). PVI times were shorter in the PFA group for all veins (median [IQR] LSPV: 5.0 [4.0–7.0] vs 10.0 [10.0–12.0] min, *p* < 0.001; LIPV: 5.0 [3.0–6.2] vs 10.0 [7.0–11.0] min, *p* < 0.001; RSPV: 5.0 [3.7–7.0] vs 10.0 [9.0–13.0] min, *p* < 0.001; RIPV: 4.5 [3.0–6.2] vs 8.0 [7.0–10.2] min, *p* < 0.001) (Table 2).

Supplemental ablation was more frequent in the PFA group: posterior wall BOX (63.3% vs 20.0%, *p* < 0.001), superior vena cava (73.3% vs 30.0%, *p* < 0.001), and mitral isthmus (40.0% vs 13.3%, *p* = 0.011) (Table 2).

### 3.4. Safety

Transient hypotension was observed in 8.3% (5/60) of patients in the PFA group, as compared to 21.7% (13/60) in the RFA group (*p* = 0.041). Transient bradycardia occurred in 5.0% (3/60) of patients in the PFA group versus 16.7% (10/60) in the RFA group (*p* = 0.040). Phrenic nerve stimulation was detected in 38.3% (23/60) of patients in the PFA group, compared with 3.3% (2/60) of those in the RFA group (*p* < 0.001) (Table 3). All episodes were transient with no persistent phrenic nerve palsy.

**TABLE 3:** Intraoperative safety outcomes.

	PFA group	RFA group	*p* value
	*n* = 60	*n* = 60	
Intraoperative adverse events			
Hypoxemia, *n* (%)	4 (6.7%)	5 (8.3%)	0.729
Hypotension, *n* (%)	5 (8.3%)	13 (21.7%)	0.041
Tachypnea, *n* (%)	8 (13.3%)	6 (10.0%)	0.570
Bradycardia, *n* (%)	3 (5.0%)	10 (16.7%)	0.040
Phrenic nerve stimulation, *n* (%)	23 (38.3%)	2 (3.3%)	< 0.001
Complications			
Cardiac tamponade	0 (0.0%)	1 (1.6%)	—
Pseudoaneurysm	1 (1.6%)	0 (0.0%)	—
Myocardial perforation	0 (0.0%)	0 (0.0%)	—
Phrenic nerve palsy	0 (0.0%)	0 (0.0%)	—
Esophageal and vagus nerve injury	0 (0.0%)	0 (0.0%)	—
Sedation‐related interventions			
Jaw thrust	1 (1.6%)	0 (0.0%)	—
Assisted ventilation	0 (0.0%)	0 (0.0%)	—
Reversal agents	0 (0.0%)	0 (0.0%)	—
Conversion to GA	0 (0.0%)	0 (0.0%)	—

*Note:* RFA, radiofrequency ablation.

Abbreviations: GA, general anesthesia; PFA, pulsed‐field ablation.

Two procedure‐related complications were documented. One cardiac tamponade occurred in a small‐stature female patient (BMI = 20.2 kg/m^2^) from the RFA group. One pseudoaneurysm was noted in an obese patient (BMI = 30.8 kg/m^2^) in the PFA group. No patient required assisted ventilation, reversal agents, or conversion to GA; only 1 patient in the PFA group required jaw thrust for transient oxygen desaturation, which resolved immediately (Table 3).

## 4. Discussion

This study was designed to explore a more streamlined conscious sedation protocol for PFA in settings with limited anesthesiologist availability. The key findings demonstrated that PFA using a variable‐loop multielectrode catheter, administered by the EP team under conscious sedation, was associated with acceptable intraoperative tolerance and safety while preserving procedural efficiency, supporting the feasibility of this approach in selected low‐risk patients.

### 4.1. Conscious Sedation: Balancing Pain, Cough, and Sedation Depth

The core goal of conscious sedation for catheter ablation is to alleviate procedure‐related discomfort while maintaining patient cooperation. This study used a midazolam–fentanyl titration regimen: Midazolam, a GABA‐A receptor modulator, provides anxiolysis and anterograde amnesia [18, 19]. Fentanyl, a *μ*‐opioid receptor agonist, provides intraprocedural analgesia. The two act synergistically, allowing lower individual doses, with titration guided by vital signs and RASS scores [20]. Under the same core sedation regimen, FPS‐R scores were lower in the PFA group, a difference attributable primarily to the ablation energy itself. RFA causes coagulative necrosis through thermal injury, and high temperatures stimulate nociceptive nerve endings in the pericardium and pleura, producing marked intraprocedural pain; PFA selectively damages cardiomyocyte membranes via irreversible electroporation without generating tissue hyperthermia, thereby avoiding thermal injury–related pain [21, 22].

Although the nonthermal mechanism of PFA reduces pain, pulsed electric fields can stimulate periairway neural plexuses and activate vagal reflexes, inducing bronchial smooth muscle spasm and cough [23]. In this study, cough was most prominent during LSPV and posterior wall BOX ablation. Anatomically, the LSPV lies adjacent to the left main bronchus, and the posterior LA wall is in close to the esophagus and vagal plexuses; the electric field generated during ablation can extend to these structures [23]. To address this, the PFA group received prophylactic triple nebulization preoperatively: Lidocaine blocks airway vagal reflexes through topical mucosal anesthesia, salbutamol relaxes bronchial smooth muscle, and budesonide attenuates mucosal inflammation and sensory nerve hyperresponsiveness [24–26]. Despite this pretreatment, cough during PVI remained more frequent in the PFA group (21.7% vs 6.7%), suggesting that cough is an inherent characteristic of PFA rather than a sign of inadequate sedation, and should be anticipated.

GA or deep sedation more completely eliminates involuntary movement and discomfort, providing stable operating conditions [27]. In this study, titration by the EP team guided by vital signs and RASS yielded comparable sedative doses and RASS scores, with balanced sedation depth. Body movement grades did not differ significantly; Grade 2 movement occurred in only 6.7% of PFA patients and 3.3% of RFA patients, and no patient required conversion to GA, indicating that the conscious sedation achieved a favorable balance between comfort and cooperation in selected low‐risk patients. Operator nonblinding may introduce observer bias in movement grading; however, FPS‐R was patient‐reported and cough was analyzed at the patient level, which partially mitigates this limitation.

### 4.2. Efficiency: Preserves and Enhances PFA’s Intrinsic Technical Advantages

Compared with RFA, PFA reduced total procedure time and LA dwell time by approximately 25%–30%. This advantage primarily reflects the intrinsic nature of PFA technology: Irreversible electroporation creates large, contiguous lesions with a single energy delivery, obviating the point‐by‐point ablation and lesion continuity verification required in RFA [28, 29]. The conscious sedation protocol did not disrupt PFA workflow, thereby preserving this technical advantage; meanwhile, by avoiding anesthesia induction and emergence, conscious sedation may further reduce procedure time [8]. Although we did not include a control arm of PFA performed under GA to directly verify this effect, comparison with published cohorts provides a reference: A recent Volt CE Mark multicenter study reported total procedure and LA dwell times of 102.1 ± 34.2 min and 40.5 ± 22.2 min, compared with 50.3 ± 10.3 min and 36.9 ± 8.6 min in our PFA group [30]. This difference may partly reflect the time saved by avoiding GA induction and emergence, although cross‐study comparisons are confounded by differences in operator experience, patient populations, lesion characteristics, and center‐specific protocols and should be interpreted with caution. Overall, conscious sedation preserved the intrinsic efficiency advantage of PFA.

### 4.3. Safety: Synergy Between PFA’s Tissue Selectivity and Anesthesia Modality

The favorable safety profile reflects the combined contribution of PFA tissue selectivity and conscious sedation. PFA acts via nonthermal irreversible electroporation, which precisely targets cardiomyocytes while sparing adjacent critical structures and avoiding energy related collateral tissue injury [31, 32]. Conscious sedation obviates complex airway procedures; it reduces GA‐related risks (respiratory depression, airway injury) and enables the real‐time assessment of patient responses via continuous communication, preventing excessive physiological suppression from deep sedation/GA [8].

Two procedure‐related complications occurred: one cardiac tamponade in the RFA group, considered related to a thin atrial wall associated with low BMI and catheter manipulation, and one pseudoaneurysm was noted in an obese patient in the PFA group; neither was directly related to the sedation protocol. Among transient adverse events, the RFA group had higher rates of hypotension and bradycardia, suggesting more vagally mediated events, possibly due to stronger autonomic stimulation from thermal injury, although the greater pain burden in the RFA group may also have triggered vasovagal responses. Phrenic nerve stimulation was more common in the PFA group, consistent with prior reports [32]. All episodes were transient, with no persistent phrenic nerve palsy, premature termination, or conversion to GA. These findings suggest that, in selected low‐risk patients, conscious sedation can support safe and stable procedure conduct. However, this study reports only periprocedural safety data and the long‐term outcomes await further evaluation.

### 4.4. Clinical Value and Resource Optimization

The generalizability of this advantage rests on the universality of the protocol itself: The core midazolam‐fentanyl titration strategy is a routine sedation regimen in EP practice, with readily available agents and a dosing logic familiar to EP teams, resulting in a low learning curve for adoption at other centers; the triple nebulization serves only as an adjunct for PFA‐related cough, and its three components are commonly used and easily administered, allowing centers to adopt or adjust them according to local resources and patient airway characteristics [33, 34]. Importantly, this study enrolled strictly selected low‐risk patients, excluding those at high anesthesia risk; therefore, the results cannot be directly extrapolated to higher‐risk AF patients, for whom GA or deep sedation remains the standard approach [35]. Our findings do not challenge current anesthesia practice but rather offer a complementary option for selected low‐risk patients in resource‐limited settings [36].

### 4.5. Study Limitations

This single‐center exploratory analysis assessed only periprocedural outcomes. The comparator was RFA under conscious sedation; therefore, the independent contribution of anesthetic modality to PFA procedural efficiency could not be isolated. In addition, the triple nebulization was administered only in the PFA group, which may confound between‐group comparisons of cough and other airway‐related outcomes. Meanwhile, operators could not be blinded to ablation modality, and outcomes such as movement grading may be subject to observer bias. The PFA group also had a higher rate of supplemental ablation; although primary pain and cough analyses were restricted to the standardized PVI phase, performance bias may persist. Furthermore, site‐specific pain during supplemental ablation was not assessed, limiting evaluation of conscious sedation adequacy beyond the PVI phase. Finally, all procedures were performed using the VARIPULSE system in selected low‐risk patients, limiting generalizability.

## 5. Conclusions

In selected low‐risk patients, EP team–administered conscious sedation is feasible for PFA, with acceptable intraoperative tolerance and safety while preserving procedural efficiency. This protocol may serve as a practical option to reduce anesthesia resource barriers for PFA in resource‐limited settings.

## Funding

This work was supported by grants from the Noncommunicable Chronic Diseases‐National Science and Technology Major Project (Grant No.: 2024ZD0521900).

## Ethics Statement

The study protocol was approved by the Ethics Committee of Fuwai Hospital, Chinese Academy of Medical Sciences (Approval No. 2024‐2570).

## Conflicts of Interest

The authors declare no conflicts of interest.

## Supporting Information

Additional supporting information can be found online in the Supporting Information section.

## Supporting information


**Supporting Information** Randomization, outcome assessment, and sedation safety protocol.

## Data Availability

The data that support the findings of this study are available from the corresponding author upon reasonable request.
