# Supplementary material for: Electrophysiology Team–Administered Conscious Sedation for Pulsed‐Field Versus Radiofrequency Ablation in Atrial Fibrillation
Source: Anesthesiol Res Pract. 2026 Oct 29;2026:4197770. doi: 10.1155/anrp/4197770 (PMC13717711; doi:10.1155/anrp/4197770)
Supplement: Supplementary file 1 [file ANRP-2026-4197770-s001.docx]

**Electrophysiology Team-Administered Conscious Sedation for Pulsed-Field versus Radiofrequency Ablation in Atrial Fibrillation**

**Supplement 1**

This Supplementary File Includes:

1. Randomization and Blinding

2. Outcome Assessments

2.1 Intraoperative patient tolerance assessment

2.2 Intraoperative ablation detailed data recording and standards

2.3 Definitions of intraoperative safety outcomes

3. Conscious Sedation Safety Protocol

**1. Randomization and Blinding**

Patients were randomized 1:1 to PFA or RFA using a computer-generated block randomization sequence (block size = 4), with the random sequence generated by an independent statistician and allocation concealed via sequentially numbered opaque sealed envelopes prepared by an independent researcher. Envelopes were opened only after written informed consent and completion of all baseline assessments. Patients and follow-up outcome assessors were blinded to group assignment; operating electrophysiologists could not be blinded due to the distinct visual differences between PFA and RFA catheters.

**2.** **Outcome Assessments**

The primary endpoint was intraprocedural pain during PVI, assessed by Faces Pain Scale-Revised (FPS-R) scores. Secondary endpoints included: (1) other tolerance outcomes; (2) procedural efficiency; and (3) periprocedural safety. The definitions and criteria are detailed in the following sections

**2.1 Intraoperative patient tolerance assessment**

Patient intraoperative tolerance was assessed using validated scales, intraoperative body movement grading, and cough reactions. RASS scores, body movement grade, and cough reactions were jointly evaluated by the primary operator and first assistant. Faces Pain Scale-Revised (FPS-R) scores obtained immediately after each pulmonary vein isolation.

**2.1.1 validated scales**

- Richmond Agitation-Sedation Scale (RASS): Used to assess the level of patient agitation and sedation, comprising 10 levels (-5 to +4). A score of -5 indicates no response to any stimulus, while +4 indicates combative behavior. The patient's response to stimuli was observed and scored to help adjust the doses of sedative and analgesic medications, balancing comfort and safety.
- The Faces Pain Scale-Revised (FPS-R): Used for the rapid assessment of pain intensity. It utilizes 6 different facial expressions ranging from "no pain" (0 points) to "most severe pain" (10 points).
- Likert Scale Questionnaire (LSQ): Consists of several declarative items that express a viewpoint on a topic. Respondents can indicate the degree to which they agree or disagree with the opinion expressed by the statement.

**2.1.2 Intraoperative body movement assessment:**

Grade 0 (no movement), Grade 1 (mild movement: slight tremor of fingers, toes, or head, no impact on catheter stability), and Grade 2 (significant movement: trunk, upper/lower limb movement, requiring procedure suspension).

Table 1 Intraoperative Body Movement Grade Criteria

| **Grade** | **Movement Performance** | **Impact on Operation** | **Intervention Measures** |
| --- | --- | --- | --- |
| Grade 0 | No non-instructive movement | No impact | No intervention |
| Grade 1 | Only slight tremor of peripheral limbs (fingers, toes) or head | Catheter position is stable, and ablation proceeds normally | No need to suspend the operation, continuous observation |
| Grade 2 | Trunk, upper limb, or lower limb movement; or attempts to pull out the catheter | Catheter position may shift, and ablation needs to be suspended | ① Verbal comfort by the nurse; ② If comfort is ineffective, add midazolam 0.5 mg (single dose) according to the sedation protocol, and restart the operation after the movement stops |

**2.1.3 Intraoperative cough assessment**

The primary between-group comparison was performed at the patient level during the standardized PVI phase, defined as at least one cough episode during PVI. Cough data from supplementary ablation sites were retained as descriptive, exploratory analysis only. A cough episode was defined as an involuntary, audible expulsive maneuver triggered by ablation energy delivery. The anatomical site of cough occurrence was recorded.

**2.2 Procedural efficiency metrics and definitions**

The following intraoperative data were recorded: total procedure time, catheter left atrial (LA) dwell time, first-pass isolation (FPI) rate and time for each pulmonary vein, and need for supplemental ablation.

Total Procedure Time: the time from skin preparation and draping until removal of all catheters and sheaths and completion of vascular compression bandaging at the puncture site(s).

- Catheter LA Dwell Time: the time from successful transseptal puncture until completion of left atrial ablation and removal of all mapping and ablation catheters from the left atrium.
- First-Pass Isolation (FPI) Rate: the ratio of pulmonary veins achieving electrical isolation after initial ablation to the total number of pulmonary veins. For the RFA group, FPI was assessed after circumferential ablation at the PV ostia without touch-up ablation. For the PFA group, FPI was assessed after the recommended protocol of 2 applications at the PV ostia and 2 applications at the PV antrum for each vein.
- First-Pass Isolation (FPI) Time: the continuous duration from the start of initial ablation to the first documentation of complete electrical isolation, verified by electrophysiology, excluding touch-up ablation and other procedural time.

**2.3 Definitions of intraoperative safety outcomes**

Intraoperative safety was assessed by documenting transient adverse events, procedural complications, and sedation-related interventions.

Patients with poor tolerance could be converted to deep sedation or general anesthesia. Flumazenil and naloxone were prepared at bedside for reversal of respiratory depression if needed.

**2.3.1 Transient adverse events**

Transient adverse events included hypotension, hypoxaemia, bradycardia, tachypnoea, and phrenic nerve stimulation. Detailed definitions are provided in Table 2.

Table 2 Definitions of Transient Adverse Events

| **Transient Adverse Event** | **Definition** |
| --- | --- |
| Transient hypotension | Systolic blood pressure <90 mmHg or >20% decrease from baseline, lasting <5 minutes |
| Hypoxaemia | SpO₂ <92% lasting >30 seconds |
| Transient bradycardia | Heart rate <50 beats per minute lasting <5 minutes |
| Tachypnoea | Respiratory rate >20 breaths per minute |
| Phrenic nerve stimulation | The definitions and criteria are detailed inDiaphragmatic contraction or hiccup observed during energy delivery, resolving immediately upon cessation of energy delivery |

**2.3.2 Procedural complications**

Procedural complications included pseudoaneurysm, cardiac tamponade, phrenic nerve palsy, myocardial perforation, and oesophageal or vagus nerve injury. All complications were diagnosed according to standard clinical criteria.

**2.3.3 Sedation-related interventions**

Sedation-related interventions included jaw thrust, assisted ventilation, administration of reversal agents (flumazenil/naloxone), and conversion to general anesthesia.

**3. Conscious Sedation Safety Protocol (For reference only)**

**3.1 Preprocedural preparation**

- All patients underwent preprocedural airway assessment (Mallampati score, mouth opening, neck mobility) and anesthesia risk stratification; patients at high anesthesia risk were excluded and referred for general anesthesia.
- Patients fasted 6 hours for solids and 2 hours for clear liquids before the procedure.
- Emergency airway equipment (bag-valve mask, oral/nasal airways, laryngoscope, endotracheal tubes) and reversal agents (flumazenil, naloxone) were prepared at bedside; a dedicated anesthesiologist was on callnasal cannula at 3 L/min and immediately available throughout the procedure.

**3.2 Intraoperative monitoring and management**

- Continuous monitoring of electrocardiogram, non-invasive blood pressure, heart rate, and pulse oxygen saturation was performed, with vital signs recorded every 5 minutes.
- Midazolam and fentanyl were titrated incrementally to a target Richmond Agitation-Sedation Scale (RASS) score of 0 to -1, with additional doses given as needed based on patient response.
- Oxygen was administered via nasal cannula at 3 L/min throughout the procedure.

**3.3 Adverse event management**

- For transient oxygen desaturation (SpO₂ <92%), jaw thrust, airway repositioning, or increased oxygen flow was performed immediately; assisted ventilation was initiated if desaturation persisted.
- For oversedation or respiratory depression, reversal agents were administered as needed, with the anesthesiologist taking over airway management immediately.
- The procedure was suspended for intolerable pain, severe agitation, or persistent adverse events, with conversion to general anesthesia if required.

**3.4 Postprocedural observation**

- All patients were monitored in the recovery unit for at least 2 hours post-procedure, with vital signs and consciousness assessed every 15 minutes until fully awake.
- Patients were discharged only after meeting standard discharge criteria (stable vital signs, no bleeding or severe pain, fully oriented).
